# Evidence for an association of HLA-DRB1*15 and DRB1*09 with leprosy and the impact of DRB1*09 on disease onset in a Chinese Han population

**DOI:** 10.1186/1471-2350-10-133

**Published:** 2009-12-11

**Authors:** Furen Zhang, Hong Liu, Shumin Chen, Changyuan Wang, Chuanfu Zhu, Lin Zhang, Tongsheng Chu, Dianchang Liu, Xiaoxiao Yan, Jianjun Liu

**Affiliations:** 1 Shandong Provincial Institute of Dermatovenereology, Jinan, Shandong Province, PR China; 2 Shandong clinical college for skin diseases, Anhui medical university, Jinan, Shandong Province, PR China; 3 Shandong blood center, Jinan, Shandong Province, PR China; 4 Genome Institute of Singapore, A*STAR, Singapore, Singapore

## Abstract

**Background:**

Human leukocyte antigens (HLAs) have been proposed to modulate the immune response to *Mycobacterium leprae*. The association of HLA-DRB1 with leprosy has been reported in several populations, but not in a Chinese population.

**Methods:**

The polymerase chain reaction-sequence-specific oligonucleotide probe with Luminex100 (PCR-SSOP-Luminex) method was used to genotype HLA-DRB1 alleles in 305 leprosy patients and 527 healthy control individuals.

**Results:**

The HLA-DRB1*15 allele was significantly more prevalent among leprosy patients than healthy controls, whereas the frequency of the HLA-DRB1*09 allele was lower among leprosy patients, especially those with early-onset disease.

**Conclusion:**

HLA-DRB1 alleles are associated with leprosy susceptibility in a Chinese population. The HLA-DRB1*09 allele was found to be protective exclusively in a subset of early-onset leprosy patients.

## Background

Leprosy is a chronic infectious disease that occurs in genetically predisposed individuals. It is caused by infection with the intracellular macrophage pathogen *Mycobacterium leprae*. Leprosy is characterized by a spectrum of disease symptoms that result from interactions between the host immune response and invading *M. leprae*. At the lepromatous extreme, patients exhibit multibacillary infection and an absence of antigen-specific cellular immunity. At the tuberculoid extreme, patients exhibit paucibacillary infection and a strong cellular immune response. Between these two extremes, borderline leprosy patients show intermediate phenotypes [1].

Leprosy was once prevalent worldwide. However, there has been a steady decline in the prevalence of leprosy since the introduction of multidrug therapy by the World Health Organization (WHO-MDT) in 1982 [2]. The detection rate of new cases, however, remains steady in some countries, including several regions of China [3,4]. Since 2002, nearly 1,600 new cases have been detected annually in China [4].

Previous studies have suggested a strong influence of genetic factors in regulation of the anti-*M. leprae *immune response [5,6]. Two recent genome-wide linkage analyses have identified chromosome 6p21 as a major leprosy-susceptibility locus [7,8]. This region harbors the HLA gene cluster, which has been extensively studied for its role in leprosy pathogenesis [9-12]. In particular, *HLA-DR *alleles have been consistently found to be associated with leprosy [13]. Several studies reported an association of the *HLA-DR2 *alleles HLA-DRB1*15, *16 *04, *10 and *12 with susceptibility or resistance to leprosy in Brazilian, Vietnamese, South Indian, Indonesian, Thai and Argentine populations [14-18]. In addition, *HLA-DR3 *alleles were also found to be associated with leprosy susceptibility in Surinamese and Mexican populations [19,20]. These studies have not only shown the importance of the *HLA-DR *locus in leprosy susceptibility, but have also highlighted the fact that different *HLA-DR *alleles are associated with leprosy in different populations.

Although leprosy remains a major public health concern and one of the infectious diseases most capable of causing disability in China, the genetic susceptibility to leprosy in Chinese populations has received little study. The only such report was an investigation of the association of *HLA-DRB1 *with leprosy in Southern China; however, due to the small sample used, this study failed to detect this well-established association [21]. In the current study, we investigated the association of *HLA-DRB1 *with leprosy by genotyping *DRB1 *alleles in a large Chinese Han sample of 305 leprosy patients and 527 healthy control individuals. We further investigated the association of *DRB1 *alleles with clinical subtype and age-of-onset of the disease.

## Methods

### Patients and controls

The study included 305 unrelated individuals with a diagnosis of leprosy and 527 control samples. The diagnosis of leprosy was based on clinical assessment and detection of acid-fast bacilli in skin-slit smears and histopathological assessment of skin lesions. The patients were further classified as multibacillary (MB) or paucibacillary (PB) on the basis of clinical and histological criteria [1]. The MB patient subset (n = 179), which included patients with lepromatous leprosy (LL), borderline lepromatous leprosy (BL)and borderline (BB) leprosy, had a bacterial index (BI) > 0. The PB group (n = 126) had a BI = 0, and included patients with borderline tuberculoid leprosy (BT) and tuberculoid leprosy (TT). Control sample donors were recruited from the Shandong Blood Center. Some characteristics of the 305 cases and 527 controls are summarized in Table 1.

**Table 1:** Characteristics of the study population of 305 cases and 527 controls

	Population	Sample size (LL/BL/BB/BT/TT) (MB/PB)	Male/Female	Mean age	Mean age onset
Case	Chinese Han	305	278/27	67.5	19.0
		(158/15/6/6/120)			
		(179/126)			
Control	Chinese Han	527	451/76	50.2	NA

NA: no onset age for controls

All subjects gave informed consent to participate in the study. The protocol was approved by the Ethical Committee of the Shandong Provincial Institute of Dermatovenereology.

### DNA extraction and HLA-DRB1* typing

Genomic DNA was extracted from peripheral blood using a commercially available DNAzol extraction kit (E.Z.N.A Blood DNA KIT, Omega Bio-tek, Inc).

The procedure for HLA genotyping by the polymerase chain reaction-sequence-specific oligonucleotide probe with Luminex100 (PCR-SSOP-Luminex) method included PCR amplification, hybridization, a streptavidin-phycoerythrin (SA-PE) reaction, and analytical measurements [22]. Target DNA was amplified by PCR using 5' biotin-labeled primers that were highly specific to certain sequences of *HLA-DRB1 *genes. PCR was carried out in a 20 μL reaction containing Lifecodes mixture (6 μL), Taq polymerase (0.2 μL), nuclease-free water (11.8 μL) and genomic DNA (3 μL). After denaturization, amplified DNA was allowed to hybridize to complementary DNA probes coupled to microbeads. The oligobead-coupled, hybridized PCR product was labeled with streptavidin-phycoerythrin. The fluorescent intensity of phycoerythrin on each coded oligobead that had hybridized with the biotin-labeled PCR product was measured using a Luminex apparatus, and the HLA-DRB1* genotype was determined with the assistance of Genosearch typing software (Quick-Type for LifeMatch 2.0, Luminex Corporation). This HLA-typing analysis was done at the Shandong blood center.

### Statistical analysis

Power calculations, carried out using PS software http://www.power-analysis.com/home.htm, showed that our sample size of 305 patients and 527 controls had greater than 95% power to detect an odds ratio (OR) of 2.0 at a significance level of 5% when the frequency of the allele of interest was greater than 0.10. Allele frequencies were calculated by direct counting. The differences in allele frequency between patients and controls and between early onset and late-onset patients were tested using logistic regression analyses with adjustments for age and gender. The significance of the allele frequency difference between the MB and PB cases was analyzed using Pearson Chi-square tests. The nominal P-values were corrected for multiple testing (Pc-value) using the Bonferroni correction (i.e., by multiplying the nominal P-values by the number of HLA alleles being tested). A Pc-value < 0.05 was accepted as statistically significant. The strength of associations was estimated by calculating ORs. Statistical analyses were carried out using SPSS software (version 11.0).

## Results

Table 2 summarizes the allele frequencies of HLA-DRB1* alleles in the leprosy patient and control populations and the results of association analyses. Of the 13 *HLA-DRB1 *alleles determined by the PCR-SSOP-Luminex method, two alleles showed a significant difference in frequency (P < 0.05) between patients and controls. The frequency of the DRB1*15 allele among patients was 0.32 (195/610), which was significantly higher than the frequency of 0.18 (185/1054) observed in the healthy controls (P < 0.001). This difference remained significant after correcting for multiple testing (Pc < 0.001). In contrast, the frequency of the HLA-DRB1*09 allele was significantly lower in the patient population than among controls (P = 0.002, Pc = 0.026).

**Table 2:** Allele frequency distribution of HLA-DRB1* in patients versus controls, and MB versus PB forms of leprosy

	Patients		Controls				MB	PB	
						
	(*n *= 305)		(*n *= 527)				(n = 179)	(n = 126)	
**Alleles**	**2 *n***	**AF**	**2 *n***	**AF**	***P*-value**	***Pc*-value**	**AF**	**AF**	***P*-value**

HLA-DRB1*01	19	0.03	23	0.02	0.246		0.02	0.04	0.136
HLA-DRB1*03	14	0.02	40	0.04	0.520		0.04	0.02	0.224
HLA-DRB1*04	44	0.07	98	0.09	0.387		0.09	0.09	0.471
HLA-DRB1*07	73	0.12	164	0.16	0.427		0.16	0.13	0.197
HLA-DRB1*08	30	0.05	53	0.05	0.965		0.05	0.04	0.497
HLA-DRB1*09	49	0.08	140	0.13	**0.002**	**0.026**	0.13	0.07	0.06
HLA-DRB1*10	5	0.01	13	0.01	0.564		0.01	0.00	0.571
HLA-DRB1*11	43	0.07	76	0.07	0.585		0.07	0.06	0.188
HLA-DRB1*12	61	0.10	114	0.11	0.879		0.11	0.12	0.705
HLA-DRB1*13	29	0.05	63	0.06	0.058		0.06	0.04	0.232
HLA-DRB1*14	31	0.05	52	0.05	0.522		0.05	0.06	0.183
HLA-DRB1*15	195	0.32	185	0.18	**< 0.001**	**< 0.001**	0.18	0.29	0.991
HLA-DRB1*16	17	0.03	33	0.03	0.844		0.03	0.03	0.838

*n*, number of individuals; AF, allelic frequency.

*P*-values are from allele-based tests.

We further investigated the association of HLA-DRB1* alleles with the clinical subtypes of leprosy. No significant differences in the allele frequencies were observed between the MB and PB forms of the disease (Table 2). We then investigated the association of HLA-DRB1* alleles with early-onset (age-at-onset ≤ 16 years; n = 141) and late-onset (age-at-onset > 16 years; n = 164) leprosy. Interestingly, whereas the DRB1*15 allele was significantly associated with both early- and late-onset leprosy, the DRB1*09 was only significantly associated with early-onset leprosy (Table 3).

**Table 3:** Allele frequency distribution of HLA-DRB1* in early versus late-onset leprosy groups

Alleles	Controls	Early onset			Late-onset		
						
	(n = 527)	(n = 141)			(n = 164)		
	AF	AF	*P*-value	*Pc*-value	AF	*P*-value	*Pc*-value
HLA-DRB1*01	0.02	0.03	0.924		0.04	0.162	
HLA-DRB1*03	0.04	0.03	0.848		0.02	0.320	
HLA-DRB1*04	0.09	0.09	0.685		0.06	0.406	
HLA-DRB1*07	0.16	0.10	0.130		0.14	0.848	
HLA-DRB1*08	0.05	0.05	0.926		0.05	0.723	
HLA-DRB1*09	0.13	0.06	**0.003**	**0.039**	0.10	0.256	
HLA-DRB1*10	0.01	0.003	0.168		0.01	0.285	
HLA-DRB1*11	0.07	0.07	0.688		0.07	0.503	
HLA-DRB1*12	0.11	0.11	0.480		0.09	0.324	
HLA-DRB1*13	0.06	0.04	0.017	0.221	0.05	0.429	
HLA-DRB1*14	0.05	0.05	0.607		0.05	0.988	
HLA-DRB1*15	0.18	0.35	**< 0.001**	** < 0.001**	0.29	**< 0.001**	**< 0.001**
HLA-DRB1*16	0.03	0.03	0.758		0.02	0.495	

*n*, number of individuals; AF, allelic frequency.

*P*-values are from allele-based tests.

All markers with allele frequency above 0.01 have been tested and were in HW equilibrium.

## Discussion

Leprosy is a chronic infectious disease caused by *M. leprae*. The disease only occurs in a small percentage (1-3%) of infected individuals [23], an observation that supports the important role of host genetic factors in the development of leprosy. The reported male-to-female patient ratio is 2:1 [24], consistent with the known role of gender as an independent risk factor for leprosy susceptibility. In our study, more male cases were collected than female cases; thus, the observed association of the *HLA-DRB1 *allele with leprosy was based on a mostly male population.

Many studies have reported an association of *HLA-DR2 *with leprosy, a linkage that is consistent with the finding that HLA-DR antigens are associated with disease and the suggestion that a majority of restriction determinants for *M*. *leprae *reside on DR, and not DP or DQ molecules [25]. A functional study of Ag-specific T-cell responses within the context of HLA-DR has shown that HLA-DR-associated immunity may be crucial in the adaptive immune response to infection [26]. Thus, HLA-DR plays a major role in the presentation of *M. leprae *antigens to T cells in leprosy patients.

By analyzing a large number of Chinese leprosy patients, our study has provided the first evidence for an association of *HLA-DRB1 *with leprosy in a Chinese population and provided additional support for the important role of HLA in the pathogenesis of leprosy. HLA-DRB1*15 has been demonstrated to be a leprosy-susceptibility allele among Brazilian [14] and Indian [15] populations. In our study, we found that HLA-DRB1*15 was also associated with an increased risk of leprosy in a Chinese population, and exhibited an allele frequency (32%) similar to that observed in Indian populations (31%) but higher than that observed among Brazilians (15%). The HLA-DRB1*09 allele was found to be associated with protection against leprosy in our study; its frequency was significantly lower in leprosy patients than in controls, and the association remained significant after correcting for multiple testing (P = 0.002, Pc = 0.026). This allele also showed a protective effect against the disease in Southern India, but the allele frequency among Indian patients was 0.017 [15], much lower than that observed in the Chinese population studied here (0.08).

It has been reported that the HLA lymphotoxin-alpha (LTA)+80 locus within the 6p21 chromosomal region is a major risk factor for early-onset (i.e., < 16 years old) leprosy [27]. To investigate whether the effect of HLA-DRB1* on leprosy risk was age-onset dependent, we stratified leprosy patients into early-onset (≤ 16 years) and late-onset (> 16 years) groups in association analyses. Interestingly, we found that HLA-DRB1*09 was associated with protection effect against early-onset leprosy (P = 0.003), but not late-onset leprosy (P = 0.285). In contrast, the HLA-DRB1*15 allele showed no age-onset-dependent effects. To our knowledge, this is the first report that HLA-DRB1*09 has an impact on disease onset. Further studies will be needed to investigate the association of the LTA+80 locus with leprosy in Chinese populations and determine whether the association at the *HLA-DRB1 *locus is independent of the LTA+80 locus.

De Vries et al. have proposed a two-step model for the development of leprosy, proposing that successful infection of *M. leprae *is first established in genetically vulnerable individuals, and subsequent clinical manifestation of the disease is influenced by additional host and environmental factors [28]. In our study, the frequencies of *HLA-DRB1 *alleles were similar in the MB and PB patient groups. This is consistent with the two-step model, suggesting that the *HLA-DRB1 *locus may influence the overall susceptibility to leprosy *per se*. It has also been reported that certain *HLA-DRB1 *alleles are associated with MB or PB forms of leprosy [29-31], suggesting that HLA-DRB1 may also be involved in the clinical manifestation of the disease.

Taken together, our association analysis of stratified patient groups indicates that the *HLA-DRB1 *locus is largely associated with leprosy *per se*, and only HLA-DRB1*09 shows an age-onset-dependent effect.

## Conclusion

In summary, by analyzing a large sample of Chinese Han leprosy patients, our genetic association study of *the HLA-DRB1 *locus provides strong evidence for an association of HLA-DRB1*15, as a susceptibility allele, and DRB1*09, as a protective allele, with leprosy in a Chinese population. Furthermore, we demonstrate for the first time that HLA-DRB1*09 has an impact on the onset of disease, exerting a protective effect only against early-onset leprosy. These alleles could act alone or in combination with other genes to confer susceptibility or resistance to leprosy in Chinese Han populations.

## Abbreviations

HLA: human leukocyte antigen; MB: multibacillary; PB: paucibacillary; PCR: polymerase chain reaction; SSOP: sequence-specific oligonucleotide probe.

## Competing interests

The authors declare that they have no competing interests.

## Authors' contributions

FZ conceived the study, participated in its design and coordination, and finalized the manuscript. HL performed experiments, collected and analyzed data, and wrote the first draft of the paper. SC was involved in study design and collection of samples. CZ was involved in performing experiments and coordination. CW, HZ, TC DL and XY participated in collecting samples. JL was involved in study design and interpretation of results, and finalized the manuscript. All authors read and approved the final manuscript.

## Pre-publication history

The pre-publication history for this paper can be accessed here:

http://www.biomedcentral.com/1471-2350/10/133/prepub
